# Detection of VAR2CSA-Captured Colorectal Cancer Cells from Blood Samples by Real-Time Reverse Transcription PCR

**DOI:** 10.3390/cancers13235881

**Published:** 2021-11-23

**Authors:** Sara R. Bang-Christensen, Viatcheslav Katerov, Amalie M. Jørgensen, Tobias Gustavsson, Swati Choudhary, Thor G. Theander, Ali Salanti, Hatim T. Allawi, Mette Ø. Agerbæk

**Affiliations:** 1 Centre for Medical Parasitology, Department for Immunology and Microbiology, Faculty of Health and Medical Sciences, University of Copenhagen and Department of Infectious Disease, Copenhagen University Hospital, 2200 Copenhagen, Denmark; sarabc@sund.ku.dk (S.R.B.-C.); tobias@sund.ku.dk (T.G.); swati@sund.ku.dk (S.C.); thor@sund.ku.dk (T.G.T.); 2 VarCT Diagnostics, Ole Maaløes Vej 3, 2200 Copenhagen, Denmark; amaliemj@sund.ku.dk; 3 Exact Sciences, Madison, WI 53719, USA; skaterov@exactsciences.com (V.K.); hallawi@exactsciences.com (H.T.A.)

**Keywords:** circulating tumor cells (CTCs), rVAR2, detection strategies, cancer, diagnostics, rare cell detection, polymerase chain reaction (PCR)

## Abstract

**Simple Summary:**

Circulating tumor cells are cancer cells that have entered blood or lymphatic vessels wherefrom they might get access to distant body parts and form metastases. The presence of cancer cells in a blood sample can be exploited for non-invasive diagnostic purposes. However, as blood consists of a vast number of healthy red and white blood cells the task of identifying the few potential cancer cells in a sample is a technical challenge. In this study we explore strategies for detecting circulating tumor cells after a pre-enrichment through binding to VAR2CSA protein coupled to magnetic beads. We evaluate the performance of a novel workflow that recognizes and detects the cancer cells based on their gene expression and compare this with the more traditional detection strategy using antibodies for cell staining. The highly sensitive assay presented here could potentially provide a novel strategy for early cancer detection.

**Abstract:**

Analysis of circulating tumor cells (CTCs) from blood samples provides a non-invasive approach for early cancer detection. However, the rarity of CTCs makes it challenging to establish assays with the required sensitivity and specificity. We combine a highly sensitive CTC capture assay exploiting the cancer cell binding recombinant malaria VAR2CSA protein (rVAR2) with the detection of colon-related mRNA transcripts (USH1C and CKMT1A). Cancer cell transcripts are detected by RT-qPCR using proprietary Target Enrichment Long-probe Quantitative Amplified Signal (TELQAS) technology. We validate each step of the workflow using colorectal cancer (CRC) cell lines spiked into blood and compare this with antibody-based cell detection. USH1C and CKMT1A are expressed in healthy colon tissue and CRC cell lines, while only low-level expression can be detected in healthy white blood cells (WBCs). The qPCR reaction shows a near-perfect amplification efficiency for all primer targets with minimal interference of WBC cDNA. Spike-in of 10 cancer cells in 3 mL blood can be detected and statistically separated from control blood using the RT-qPCR assay after rVAR2 capture (*p* < 0.01 for both primer targets, Mann-Whitney test). Our results provide a validated workflow for highly sensitive detection of magnetically enriched cancer cells.

## 1. Introduction

Colorectal cancer (CRC) is the second leading cause of cancer death worldwide [1]. Overall survival rates are highly dependent on the advancement of the disease at the time of diagnosis, which emphasizes the importance of early detection. The concept of ‘liquid biopsies’, such as blood samples containing circulating tumor cells (CTCs), circulating tumor DNA (ctDNA) or other tumor-derived components have gained increasing attention for the management of patients with CRC [2]. CTCs are cells that have left the tumor site and entered the blood circulation from where they may give rise to distant metastases or local relapse [3]. Previously, the presence of CTCs in the bloodstream was mainly measured in late-stage cancer patients. However, more recent studies have shown detection of CTCs in peripheral blood of early-stage cancer patients [4,5], including patients with colorectal adenomas [6,7]. CTCs are extremely rare compared to the excessive number of white blood cells (WBCs) in a blood sample, especially in the case of early stage cancer, making efficient and specific isolation challenging [8]. Furthermore, CTC numbers in CRC patient blood samples are even below what is found within other carcinomas such as breast and prostate cancer [9]. Thus, in order to enable CTC analysis in a broad group of CRC patients, there is an urgent need for more sensitive approaches.

A plethora of CTC isolation strategies exist, most of which comprise of two main steps: capture or enrichment of the cancer cells followed by detection through either staining with fluorescently labeled antibodies or nucleic acid identification by polymerase chain reaction (PCR) methods [10]. In order to increase the sensitivity of a CTC capture assay, both of these steps must be optimized for efficient and sensitive identification of target cells. We have previously shown that the recombinant malaria-derived VAR2CSA protein (rVAR2) effectively and specifically binds cancer cells of various tissue origin by interacting with a unique oncofetal chondroitin sulfate (ofCS) displayed on the cell surface [11,12]. Based on this, we developed a CTC assay exploiting the cancer cell binding property of rVAR2 for CTC capture from several types of cancer patient blood [13,14]. We have further optimized the workflow, which has recently shown to enable capture of colorectal cancer cells from whole blood samples with increased sensitivity [15]. The aim of this study is to explore the sensitivity and specificity of detection strategies downstream of rVAR2-capture by comparing antibody-based detection of cytokeratin (CK) with the measurement of colon-related mRNA transcripts by reverse transcription PCR (RT-PCR).

We apply the long-probe quantitative amplification signal (LQAS) technology, which is an evolution of the QuARTs technology, that has previously been developed and validated for detection of aberrantly methylated genes [16,17]. This technology combines the polymerase-based DNA amplification with an invasive probe cleavage-based signal amplification process, allowing accumulating number of cleaved probes to generate fluorescence in each cycle [18]. Thereby, the LQAS technology adds a linear amplification signal to the overall reaction, increasing the sensitivity of the assay [16]. In addition, we add a target enrichment step (TE) to the reverse transcription reaction by including 12 cycles of amplification using target-specific primers. Our results demonstrate that the combination of highly sensitive rVAR2 cell capture and downstream detection of colon-related mRNA transcripts by RT-qPCR provide a beneficial workflow for the identification of rare colorectal cancer cells in blood.

## 2. Materials and Methods

### 2.1. Protein Production and Biotinylation of rVAR2

Recombinant VAR2CSA (rVAR2) with an N-terminal SpyTag (SpyT) was produced in SHuffle^®^T7 Competent *E. coli* cells as previously described [14]. Protein was purified to homogeneity and characterized for CS-specific binding by ELISA and flow cytometry as previously described [15]. SpyCatcher (SpyC) was produced in the *E. coli* BL21 strain and subsequently multibiotinyalted using NHS-biotin (Sigma-Aldrich, MERCK, Darmstadt, Germany) as previously described [14]. SpyT-rVAR2 and SpyC-biotin were mixed in a 0.8:1 molar ratio and left at room temperature for 1 h to allow formation of a covalent isopeptide bond between the Spy-tag and the SpyC [19].

### 2.2. Cell Culture

COLO205 (ATCC# CCL-222™) and SW480 (ATCC# CCL-228™) cells were cultured in RPMI 1640 medium with GlutaMax™ (Sigma Aldrich), supplemented with 10% fetal bovine serum (FBS), penicillin, and streptomycin. Cells were kept at 37 °C and 5% CO_2_. The fraction of COLO205 cells growing in suspension was used for spike-in experiments, while adherent SW480 cells were detached using CellStripper^®^ solution for 10 min. To validate capture efficiency independently of detection method, cells were stained with CellTracker™ (Green CMFDA or Orange CMRA) dye (Thermo Fisher Scientific, Waltham, MA, USA) according to manufacturer’s instructions prior to spike-in to blood. Validation of the number of cells spiked into blood samples was done as previously described [15].

### 2.3. Magnetic Bead Preparation

Sera-Mag™ SpeedBeads Streptavidin-Blocked Magnetic Particles (Cat. no. 21152104010150, GE Healthcare, Chicago, IL, USA) were washed and blocked in Pierce^TM^ Protein-Free (PBS) Blocking Buffer (PF) (Cat. no. 37572, Thermo Fisher Scientific) for at least 1 h prior to cell capture. Beads were 6-fold diluted in blocking buffer before use.

### 2.4. Cancer Cell Capture from Blood

Blood samples were drawn from healthy donors using K2 EDTA Vacutainer^®^ blood collection tubes and processed within 2 h. Cancer cells were spiked into 3 mL of blood, followed by the addition of 27 mL RBC lysis buffer reaching a final concentration of 0.155 M NH_4_Cl, 0.01 M KHCO_3_, and 0.1 mM EDTA and incubated at RT for 13 min. After centrifugation (400× *g* for 8 min) the pellet was washed once in Dulbecco’s PBS (DPBS) (Cat. no. D8537, Sigma) and finally resuspended in 300 µL DPBS supplemented with 5% FBS. Biotinylated SpyC-SpyT-rVAR2 conjugate was added to the cell suspension to reach a final concentration of 100 nM, and samples were incubated for 30 min at 4 °C with slow rotation. Subsequently, samples were spun at 350× *g* for 5 min. and cell pellets were gently resuspended in 600 µL DPBS with 0.1% BSA and 2 mM EDTA (or PF buffer supplemented with 1 mM EDTA in case of immunocytochemical staining). 25 µg of beads were added to each sample followed by incubation for 30 min at 4 °C with slow rotation. Bead-bound cells were captured by placing sample tubes in a magnet rack for 2 min. and used for immunocytochemistry (Section 2.5) or RNA extraction (Section 2.6).

### 2.5. Cancer Cell Detection by Immunocytochemistry

Samples were immediately fixed with cold 4% PFA (Cat. no. J61899.AK, Alfa Aesar, Haverhill, MA, USA) for 10 min. Cells were incubated with FITC-conjugated pan-CK antibody (Clone: CK3 6H5, dilution: 1:50, Cat. no. 130-118-964, Miltenyi Biotec, Lund, Sweden), PE-labelled anti-CD45 antibody (Clone: 5B1, dilution: 1:10, Cat. no. 170-078-081, Miltenyi Biotec), and PE-labelled anti-CD66b antibody (Clone: REA306, dilution: 1:50, Cat. no. 130-122-922, Miltenyi Biotec) in a DPBS-based buffer supplemented with 0.01% saponin and 5% FBS for 20 min. at room temperature. Additionally, cells were stained with DAPI (Cat. no. D1306, Thermo Fisher Scientific) to visualize cell nuclei. Finally, samples were resuspended in 70 µL DPBS and transferred to a 24-well glass bottom Sensoplate™ (Cat. no. 662892, Greiner Bio-One, Kremsmünster, Austria). The plate was placed on a magnet, and DPBS was removed prior to mounting of the samples. All samples were scanned for the DAPI, FITC, and PE signal using the Cytation™ 3 Cell Imaging Multi-Mode Reader with a 10X objective. Scans were manually analyzed for the presence of FITC+, PE−, DAPI+ hits which were regarded as cancer cells if appearance also resembled a cell-like morphology in bright field settings.

### 2.6. Cell Lysis and RNA Extraction

Upon cancer cell capture (as described in Section 2.4), bead-bound cells were lysed in 3.2 M guanidine thiocyanate, 7.5% IGEPAL^®^ CA-630, 25% Isopropanol. After thorough mixing, beads were removed by magnetic force. Alternatively, it was explored whether the lysis step would benefit from removal of beads prior to lysis. This was done by adding 50 µL 1X Trypsin-EDTA solution (0.05% trypsin, 0.02% EDTA) to the bead-bound cells, followed by an incubation for 10 min. on ice, upon which the sample was placed on a magnet for 2 min. and the supernatant containing released cells was transferred to a clean tube and lysed.

Samples were either stored in lysis buffer at −80 °C or immediately processed for RNA extraction. RNA purification was performed using the QIAamp Circulating Nucleic Acid Kit (Cat. no. 55114, Qiagen, Copenhagen, Denmark) according to manufacturer’s instructions. In brief, cell lysates were applied to the columns, and membranes with bound nucleic acids were dried for 10 min. at 56 °C followed by elution of adsorbed material in 55 µL elution buffer. Eluted RNA was kept on ice and immediately processed to cDNA.

### 2.7. Validation of Organ-Specific RT-qPCR Assays

USH1C and CKMT1A genes with high and specific expression in transverse colon tissue and in COLO205 cell line were selected for this study. Both genes are not expressed in any types of blood cells according to The Human Protein Atlas (https://www.proteinatlas.org/, accessed on 22 November 2021) and Expression Atlas (https://www.ebi.ac.uk/gxa/home, accessed on 22 November 2021) databases. CASC3 gene (NCBI Reference Sequence: NM_007359.5) was selected as a control uniformly expressed in most of the tissues. PCR primers for USH1C mRNA (NCBI Reference Sequence: NM_005709.4) detection were designed to target exon 2 and exon 3 junction, while CKMT1A mRNA (NCBI Reference Sequence: NM_001015001.2) was targeted at exon 7 and exon 8 junction to eliminate cross-reactivity with genomic DNA. Sequence-specific mediator probes spanning exon/exon junction and corresponding FRET cassettes were of proprietary designs in accordance with LQAS technology. Validation of assays was performed on RNA extracted from healthy tissue derived from stomach (Cat. no. 636578, Takara, Kusatsu, Shiga, Japan), colon (Cat. no. R1234090-50, BioChain, Newark, CA, USA), esophagus (Cat. no. R1234106-50, BioChain), liver (Cat. no. 636531, Takara), lung (Cat. no. 636524, Takara), ovary (Cat. no. R1234183-10, BioChain), pancreas (Cat. no. 636577, Takara), prostate (Cat. no. 636550, Takara), uterus (Cat. no. 636551, Takara), breast (Cat. no. R1234086-50, BioChain), and white blood cells (WBCs, RNA extraction was performed from buffy coats using the QIAamp RNA Blood Mini Kit, Cat. no. 52304, Qiagen). In addition, RNA extracted from COLO205 cells and human genomic DNA (Cat. no. 69237, Novagen, MERCK, Darmstadt, Germany) were included. The assay was performed in a 1-step RT-qPCR reaction. Each reaction contained 200 nM of each forward and reverse primer, 500 nM of each FRET cassette and detection probe, 250 µM dNTPs (combined to a total volume of 3 µL), 0.5 µL of 1:10 diluted M-MLV Reverse Transcriptase (Cat. no. M1701, Promega, Madison, WI, USA), 1.5 µL 20× enzyme mix containing both GoTaq^®^ DNA polymerase (Promega) and Cleavase^®^ (Roche, Basel, Switzerland) with all buffer components, 10 µL of sample material and 15 µL nuclease-free water resulting in a total reaction volume of 30 µL. The multiplex RT-qPCR reaction was performed in the LightCycler^®^ 480 Instrument II (Roche) using the following cycling conditions: reverse transcription at 42 °C for 30 min., denaturation at 95 °C for 3 min., 45 cycles of 95 °C for 20 s, annealing at 63 °C for 60 s, and extension at 70 °C for 30 s with signal acquisition at the end of the annealing step.

### 2.8. Cancer Cell Detection by Target Enriched RT-qPCR

Synthesis of cDNA and pre-amplification of target sequences were performed using the M-MLV Reverse Transcriptase (Cat. no. M1701, Promega) and GoTaq^®^ Hot Start Polymerase (Cat. no. M5001, Promega) combined with dNTPs and target specific primers as described above in a total volume of 25 µL to which 50 µL of purified sample RNA was added. The reverse transcription was carried out at 42 °C for 30 min. followed by a pre-incubation at 95 °C for 5 min. and 12 amplification cycles consisting of 30 s at 95 °C and 60 s at 64 °C. Healthy colon RNA was always included as a positive control and nuclease-free water were included as a no target negative control for the RT-PCR reaction. The pre-amplified product was stored at −20 °C or immediately processed for qPCR analysis.

Initially the pre-amplified product was 10-fold diluted in nuclease-free water. Multiplex qPCR was performed targeting USH1C (on FAM channel), CKMT1A (on HEX channel) and CASC3 (on CY5 channel) using the LQAS technology. The amplification reaction mixture contained 200 nM of each forward and reverse primer, 500 nM of each FRET cassette and detection probe, 250 µM dNTPs (combined to a total volume of 3 µL), 1.5 µL enzyme mix containing both GoTaq^®^ DNA polymerase (Promega) and Cleavase^®^ (Roche), as well as buffer components, 10 µL of pre-amplified and diluted sample material and 15.5 µL nuclease-free water resulting in a total reaction volume of 30 µL. The multiplex qPCR reaction was performed in the LightCycler^®^ 480 Instrument II (Roche) using the following cycling conditions: denaturation at 95 °C for 3 min, 45 cycles of 95 °C for 20 s, annealing at 63 °C for 60 s, and extension at 70 °C for 30 s with signal acquisition at the end of the annealing step. Calibrator DNA derived from plasmids enzymatically digested to obtain a linearized product containing the target sequence was included in each run. Calibrator plasmids were ordered from Genewiz, and Poisson quantified by PCR for exact copy number. In order to generate a standard curve, calibrators were prepared in a series of 10-fold dilution ranging from 200,000 to 20 strands per reaction. No template controls (NTC) were also included in each run.

### 2.9. Analysis of qPCR Data Using the LightCycler^®^ 480 Software

Absolute quantification of strand count in each reaction was performed using the LightCycler^®^ 480 software analysis module ‘Absolute Quantification/Second Derivative Maximum’. Calibrators were included in duplicates in each reaction and served as internal standard curve for quantification. In addition, each amplification plot was examined manually in order to identify abnormal plots. Valid amplification plots were defined as consisting of a linear baseline region, followed by a phase of exponential amplification and finally reaching a plateau [20].

## 3. Results

### 3.1. Cytokeratin Antibodies Provide a Sensitive Strategy for Cancer Cell Detection after rVAR2 Capture

**Figure 1:**
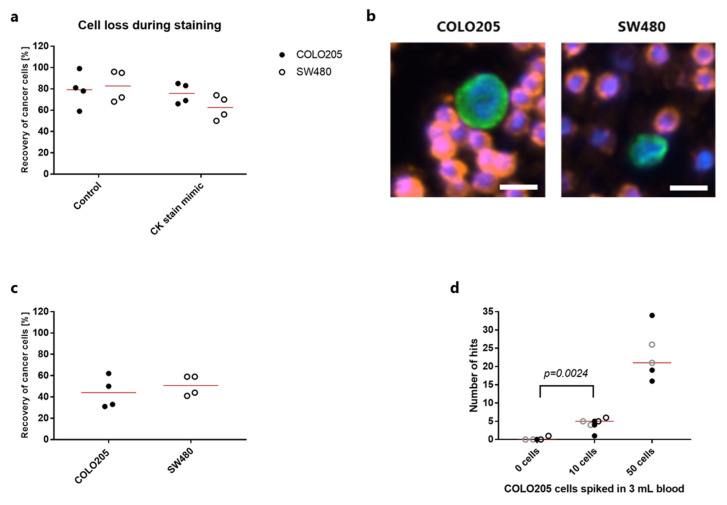
Detection of colorectal cancer cells by rVAR2 capture and immunocytochemistry. (**a**) Recovery (in percentage) of 100 CellTracker pre-stained COLO205 cells (closed circles) or SW480 cells (open circles) after spike-in to 3 mL blood and recovered with rVAR2 through binding to magnetic SeraMag beads. CK stain mimic samples were subjected to a complete staining protocol, but without any antibodies added. Lines represent the mean. (**b**) Representative images of a COLO205 cell (left) and a SW480 cell (right) stained with FITC-labeled anti-Cytokeratin antibody (green). Cell nuclei were stained with DAPI (blue) and white blood cells were stained with PE-conjugated antibodies targeting CD45 and CD66b (red). Scale bar, 10 µm. (**c**) Recovery (in percentage) of 100 COLO205 or SW480 cells after spike-in to 3 mL blood and recovered with rVAR2 through binding to magnetic SeraMag beads. Cancer cells were stained with antibodies as in (**b**) and identified as CK+, CD45/CD66b−, DAPI+ hits. Lines represent the mean. (**d**). Absolute number of CK+, CD45/CD66b−, DAPI+ hits found after rVAR2 capture from 3 mL blood spiked with 50, 10 or 0 COLO205 cells followed by staining with antibodies as in (**b**). Each type of symbol represents an individual experiment (*n* = 3 for 0 and 10 cell spike-in, *n* = 2 for 50 cell spike-in) always carried out in duplicates or triplicates. Lines represent median. 0 cells vs 10 cells were compared statistically using a Mann-Whitney test.

Our previously published data have shown an efficient capture of the COLO205 and SW480 colorectal cancer cell lines when spiked into 3 mL whole blood [15]. In that study, cells were prestained with CellTracker dye, enabling detection independently of staining procedure and marker expression. However, when adding a CK-based detection step downstream of the cell capture, the cancer cell recovery might decrease due to additional handling steps or inefficient CK staining. To assess the effect of the staining procedure, we prestained COLO205 and SW480 cells with CellTracker and spiked 100 cells into 3 mL blood. Following capture and fixation, half of the samples underwent a “CK stain mimic” procedure without any antibodies. The result showed a tendency of cell loss for SW480 cells upon application of the staining protocol from a mean recovery of 82.8% for control to 62.5% for stain mimic, while COLO205 cells seemed relatively unaffected (mean recovery of 79% for control and 76% for stain mimic) (Figure 1a).

Different CK expression levels could also affect the sensitivity of cell detection. Both colorectal cancer cell lines were positive for cytokeratin when stained with a pan-CK antibody after rVAR2-based bead capture (Figure 1b). However, when combining the rVAR2 capture and CK detection, COLO205 or SW480 cells only showed an overall recovery of 44% and 50.8%, respectively (Figure 1c). The sensitivity and specificity were assessed by spiking 50, 10 or 0 COLO205 cells into blood (Figure 1d). The assay consistently enabled detection of 50 COLO205 cells. Blood samples containing 10 COLO205 cells could be distinguished from healthy control samples with no cells spiked in (*p* = 0.024, Mann-Whitney test) (Figure 1d). Among these unspiked samples, only one replicate contained a single CK+, CD45/CD66b−, DAPI+ hit with a cell-like morphology (Figure 1d).

### 3.2. USH1C and CKMT1A Are Specifically Expressed in Intestinal-Related Tissues and Cancer Cell Lines

**Figure 2:**
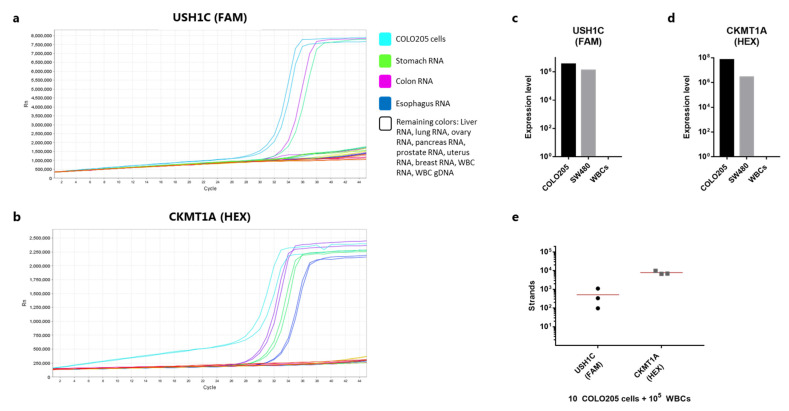
Validation of RNA markers for cancer cell detection. (**a**) Amplification curve showing the RT-qPCR reaction (one step) using primers and probes targeting USH1C (detected in the FAM channel). Samples tested include lysed COLO205 cells, RNA derived from a range of human tissues (explained in the color legend), and genomic DNA from white blood cells. All samples were tested in duplicates. (**b**) As in (**a**), but with primers and probes targeting CKMT1A (detected in the HEX channel). (**c**) Number of detected strands of USH1C cDNA from 1000 lysed COLO205 or SW480 cells measured by qPCR after RNA extraction, RT-PCR and pre-amplification. 100,000 WBCs were included in the procedure as a negative control. (**d**) As in (**c**), but showing the number of CKMT1A strands. (**e**) Number of cDNA strands of USH1C or CKMT1A detected from 10 COLO205 cells spiked in 100,000 white blood cells and directly lysed (n = 3). RNA was extracted and cDNA converted before being quantified by qPCR. Lines represent the mean.

An alternative approach for detection is to lyse the captured cells and detect the presence of tumor or tissue-specific RNA transcripts by polymerase chain reaction (PCR). To investigate the use of this method following magnetic capture of rVAR2-bound cells, we sought for relevant mRNA targets which would be present in cells of colorectal origin, but with no or minimal expression in healthy white blood cells (WBCs). Based on healthy tissue RNA-seq analysis covering several human body compartments, two organ-specific RNA targets predicted to be expressed in colon tissue were selected (USH1C and CKMT1A). In addition, a control gene ubiquitously expressed by all human cells (CASC3) was included. Primers, FRET cassettes and detection probes were designed for all three targets. The colon-specific genes were validated by assessing expression levels in RNA extracted from a broad range of organ sources: stomach, colon, esophagus, liver, lung, ovary, pancreas, prostate, uterus, and breast as well as RNA from WBCs and COLO205 cells. Among all these sources, USH1C was shown to be expressed by COLO205 cells as well as in stomach and colon tissue (Figure 2a). CKMT1A was expressed in stomach, colon, and esophagus tissue and by COLO205 cells (Figure 2b).

As a negative control, human genomic DNA was also included. Primers were designed to span an exon-exon junction, and in line with this none of the primer pairs gave rise to amplification products from genomic DNA. Finally, we compared the expression levels of the two genes in COLO205 and SW480 cells as well as in WBCs. For both genes COLO205 cells showed a considerably higher expression compared to SW480 (Figure 2c,d). The expression level of the control gene was also markedly higher for COLO205 cells compared to SW480 cells (Figure S1), indicating an overall more active mRNA synthesizing cell phenotype. Notably, both cell lines expressed more of CKMT1A compared to USH1C (Figure 2c,d). No target gene amplification was observed for the WBC sample, while all three types of cell sources showed expression of the CASC3 control gene (Figure S1). To investigate, whether the workflow from RNA extraction to qPCR would allow for robust detection of low numbers of cancer cells in a background WBCs, we prepared triplicate samples each containing 10 COLO205 cells spiked directly in 100,000 WBCs. The cells were immediately lysed and processed for RNA purification etc. Detection of 10 COLO205 gave a high variance in strand count of the USH1C gene (mean: 511, CV: 103%) (Figure 2e). From the same samples, the expression of CKMT1A was somewhat more consistent (mean: 7820 strands, CV: 22%) (Figure 2e). However, for all three samples 10 cells were repeatedly detectable.

### 3.3. Analytical Validation of RT-qPCR Assay Performance for Detection of Colorectal Cancer Cells

**Figure 3:**
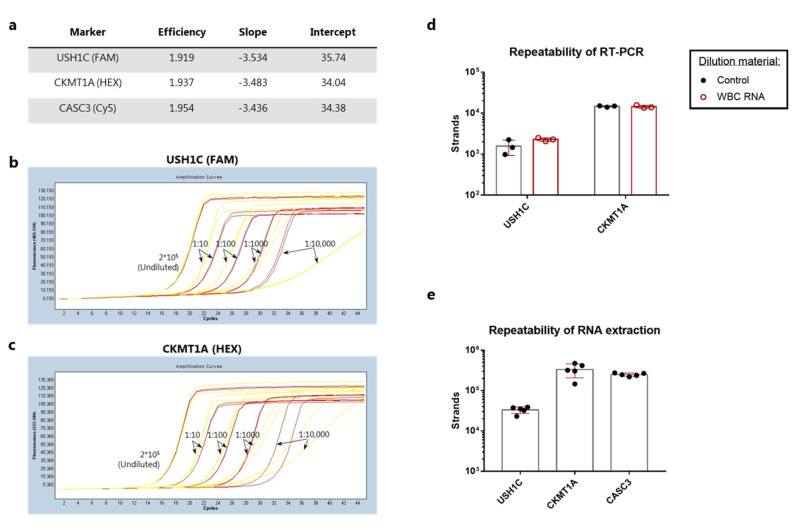
Analytical validation of the qPCR, RT-PCR and RNA extraction procedure. (**a**) qPCR assay performance on linearized calibrator sequences. A standard curve was generated from duplicate samples containing 20–200,000 strands per reaction (10-fold dilutions) and analyzed in the LightCycler^®^ software by plotting Cp values as a function of log(conc). For each primer pair the efficiency of amplification, the slope of the standard curve as well as the intercept at log(1 strand) is shown in the table. (**b**) Calibrator dilutions using either TE buffer with carrier RNA (red curves) or cDNA generated from WBCs (yellow curves). Calibrators were detected with the USH1C primers. (**c**) Same as in (**b**), but with detection using the CKMT1A primers. (**d**) Triplicate samples of 0.1 ng pre-purified human colon RNA diluted in buffer (control, black circles and bars) or WBC RNA (red circles and bars) prior to RT-PCR. (**e**) RNA purification from five samples containing 1000 COLO205 mixed with 50,000 white blood cells using separate QIAamp spin columns. Bars represent the mean strand count and error bars show standard deviation in (**d**,**e**).

We aimed to validate the RT-qPCR assay performance at individual steps as suggested by the Minimum Information for Publication of Quantitative Real-Time PCR Experiments (MIQE) guidelines [21]. This was done in the opposite order of the assay workflow, starting with the qPCR reaction and then adding each step sequentially. First, we sought to validate the primer efficiency and assess the limit of detection (LOD) for the qPCR reaction based on the LQAS technology. Calibrator DNA at a stock concentration of 20,000 strands/µL was diluted 10-fold in TE buffer containing carrier RNA. The dilution was repeated into a series of 4 dilutions with the last dilution theoretically containing 2 strands/µL. Ten µL of each calibrator were run in duplicate reactions. The LightCycler^®^ software was utilized for the generation of standard curves showing the crossing point (Cp) value as a function of log(Conc.) (Figure S2), and amplification efficiency showed near perfect efficiency (E~2) for all target genes (Figure 3a). To extend this, we also tested the efficiency and sensitivity of the qPCR reaction when diluting the calibrators in cDNA from WBCs. This was done to mimic a CTC capture sample, where a number of WBCs inevitably would be present in the bead pull-down together with the target cells. The presence of WBC cDNA resulted in successful calibrator amplification curves with Cp values in close proximity to the corresponding control calibrator concentration for all dilutions except the lowest concentration of 2 strands/µL (Figure 3b,c). At this concentration the presence of WBC cDNA resulted in lack of successful amplification curve for USH1C primers and a delayed amplification for the CKMT1A primers (Figure 3b,c).

Next, we assessed the repeatability of the RT-PCR reaction using pre-purified human colon RNA. While the CKMT1A gene showed a CV value of 4%, the intra-assay variance for USH1C was markedly higher with a CV at 41% (Figure 3d). To further validate the RT-PCR conditions, we investigated whether the presence of WBC RNA could have any inhibitory effect on the reverse transcription of target genes. When colon RNA was diluted in freshly purified WBC RNA a slight increase in strand count of USH1C was observed along with a reduction in CV value to 9% (Figure 3d). No difference could be observed for detection of CKMT1A expression (Figure 3d). Altogether, this indicated that the presence of WBC RNA was not inhibiting the RT-PCR reaction.

Finally, we evaluated the RNA extraction step. The intra-assay variance between five identical samples containing 1000 COLO205 cells mixed with 50,000 WBCs was measured after cell lysis and RNA purification. The number of target sequences within each sample revealed coefficients of variation (CV) at 19% for USH1C, 38% for CKMT1A, and 9% for the control gene (Figure 3e).

### 3.4. Detection of rVAR2-Captured Colorectal Cancer Cells by RT-qPCR

**Figure 4:**
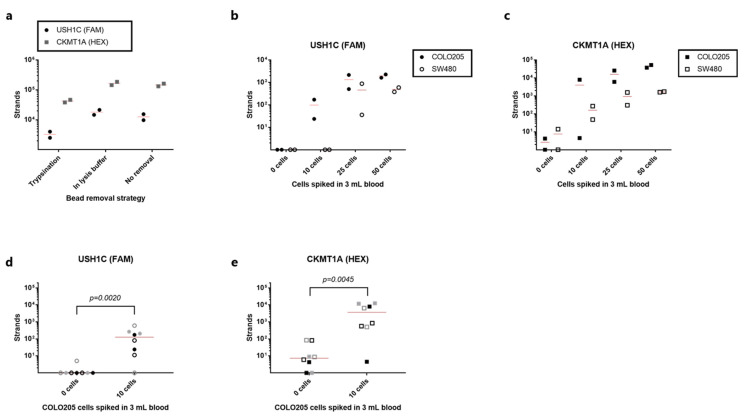
Detection of rVAR2-captured cancer cells by RT-qPCR. (**a**) cDNA strand count for USH1C (black circles) and CKMT1A (grey squares) detected after capture of 100 COLO205 cells from 1 mL blood using the rVAR2-approach. Beads were either removed prior to cell lysis by trypsinization, removed directly from the lysed product or left in the lysed product (no removal). Lines represent the mean. (**b**) cDNA strand count for USH1C detected after spike-in of 50, 25, 10 or 0 COLO205 cells (closed circles) or SW480 cells (open circles) to 3 mL blood (each test was performed in duplicate). Cells were captured with rVAR2 via magnetic beads, and RNA transcripts were quantified by RT-qPCR. Lines represent the mean. (**c**) As in (**b**) but for CKMT1A. (**d**) Number of detected cDNA strands of USH1C after rVAR2 capture of 10 or 0 cells from 3 mL blood. Each type of symbol represents an individual experiment (n = 4) always carried out in duplicates. Data from the COLO205 experiment shown in (**b**,**c**) is included as a 4th replicate. Lines represent median. 0 cells vs 10 cells were compared statistically using a Mann-Whitney test. (**e**) As in (**e**) but for CKMT1A.

While the validation of the RNA extraction, RT-PCR and qPCR reaction showed assay performance with an acceptable degree of repeatability, efficiency and sensitivity, this was so far only tested on cell mixtures without prior bead-based enrichment. Thus, it was initially tested whether the rVAR2-coated SeraMag™ magnetic beads would interfere with subsequent RNA extraction by: 1. removing the beads by trypsinization prior to cell lysis, 2. removing the beads after a direct lysis of bead-bound cells, or 3. leaving the beads in the sample after cell lysis. The three strategies were tested on rVAR2-captured samples of 100 COLO205 cells spiked into 1mL blood. An increased number of detected strands could be observed for both gene targets when removing the beads in lysis buffer compared to the trypsin-based removal (Figure 4a). The number of USH1C transcripts increased from an average of 3260 strands to 18,050 strands (5.5 fold increase), whereas CKMT1A copies increased from 42,300 strands to 165,500 strands (3.9 fold increase). For USH1C there was a reduction in mean strand count from 18,050 strands to 12,585 when leaving the beads in the lysate for RNA extraction as compared to removal of beads prior to transferring the sample to the RNA purification column (Figure 4a). The same trend was observed for CKMT1A where the mean strand count went from 165,500 to 147,000. Thus, beads were removed from the lysed product before RNA purification in the following experiments.

In order to assess the sensitivity and specificity of the entire workflow from cancer cell capture to detection by qPCR, duplicate samples of 3 mL blood were spiked with 50, 25, 10, or 0 cells. USH1C transcripts were consistently detected from both SW480 and COLO205 cells when spiking in 50 or 25 cells (Figure 4b). Furthermore, 10 COLO205 cells could be detected by targeting USH1C mRNA, whereas none of the SW480 cell samples at such low cell concentration were positive for this target (Figure 4b). No amplification of USH1C target sequence was observed in any of the unspiked samples. When targeting CKMT1A, 25 and 50 cells were easily detected for both cell lines (Figure 4c). However, the samples with no added cancer cells also showed low-level expression of this marker. Nonetheless, cancer cell detection by the expression of CKMT1A proved to be a more sensitive strategy for SW480 detection than USH1C, since down to 10 cells were efficiently detected (Figure 4c). A high variation was observed in the HEX-channel, representing the CKMT1A gene, in the samples spiked with 10 COLO205 cell. Here, 8000 strands were detected in one sample, whereas only 4.5 strands were detected in the other duplicate (Figure 4c). The linear quantification range of the calibrators spans from 200,000 to 20 strands/reaction, and therefore any strand count below 20 would technically be regarded as negative. The expression of the control gene was detected in all samples confirming consistent RNA extraction, cDNA generation and amplification (Figure S3).

To further investigate the ability of the entire workflow to detect 10 COLO205 cells in 3 mL blood, we performed three independent experiments, each with duplicate samples. Six out of eight samples were positive for USH1C mRNA, while seven out of eight samples were positive for the CKMT1A gene (Figure 4d,e, respectively). In terms of specificity, none of the unspiked samples had detectable levels of USH1C mRNA (>20 strands), while two of the healthy control samples were positive for CKMT1A. Notably, the two negative control samples with detectable transcript levels were derived from two different blood donors. Nevertheless, for both gene targets the samples with 10 cells spiked in 3 mL gave a significantly higher signal than samples without cancer cells (*p* = 0.0020 and 0.0045 for USH1C and CKMT1A, respectively, Mann-Whitney test), indicating that the complete assay was able to differentiate between a sample with only few cells and negative sample.

## 4. Discussion

The aim of this study was to investigate whether measurement of colon-specific transcripts after rVAR2-based magnetic capture of colorectal cancer cell lines would enable an alternative strategy for target cell detection compared to immunocytochemical staining using an anti-cytokeratin (CK) antibody. Here, we show proof of concept that molecular analysis of tissue-specific transcripts can be performed after rVAR2-capture of cancer cells from blood. We perform an analytical validation of the workflow including separation of nucleic acid from beads used for capture, RNA purification using QIAamp Circulating Nucleic Acid Kit, reverse transcription of mRNA into cDNA, and finally detection using the LQAS technology in a qPCR setup. Importantly, this enabled detection of cancer cells down to 10 cells in 3 mL, demonstrating a high degree of sensitivity. In spite of this, the superiority of this detection strategy over CK-based cancer cell staining could not be confirmed with the data presented here. In previous studies rVAR2 has demonstrated robust capture of as few as 3 cells in 5 or 3 mL blood [13,15]. However, as those studies were utilizing pre-stained or GFP-expressing cancer cells for spike-in, it would be interesting for future studies to challenge the sensitivity in larger blood volumes when combined with either of the two detection workflows described in this article.

It is important to underline that the two targeted genes, USH1C and CKMT1A, were chosen based on their selective expression in gastrointestinal tissue. Thus, it remains to be investigated whether these markers are expressed in colorectal tumor tissue and in clinical CTCs, and whether changes in expression levels occur during therapy. Nonetheless, these markers were chosen to demonstrate the analytical feasibility of this approach, and other CTC-relevant markers can easily be incorporated into the optimized assay.

The most widely utilized CTC detection strategy is based on immunocytochemical staining of intact, captured cells with antibodies targeting intracellular CK. However, there are several drawbacks of using this approach. First, expression of this epithelial marker has been shown to be affected by phenotypic changes, such as epithelial-to-mesenchymal transition (EMT), which is thought to play a major role during the metastatic cascade [22,23]. Low level expression of CK might not reach the threshold for detection using standard scanning microscopy for analysis. Furthermore, additional handling steps and the staining procedure itself might have an impact on the final assay sensitivity. In this study, we show that the recovery of colorectal cancer cells from the SW480 cell line is negatively affected by the staining procedure (Figure 1a). Another study addressed the same issue using CAPAN-1 pancreatic cancer cells spiked into 10^8^ peripheral blood mononucleated cells and also found a considerable reduction in recovery upon staining after EpCAM-based capture [24]. Furthermore, we observed a drop in recovery when moving from pre-stained cancer cells subjected to a stain mimicking protocol to actually detecting the spiked cells by their CK positivity (Figure 1c). This indicates that some cells are not being efficiently stained, or that magnetic beads perhaps are covering the cells and thereby complicating their detection. Altogether, this data points to the fact that sensitivity of the assay could be compromised by the staining procedure.

An alternative detection strategy is to detect cancer-specific mRNA transcripts in the bulk sample after magnetic capture [25]. In this study, we have established and analytically evaluated the detection of mRNA transcripts from cancer cells after capture with rVAR2 coupled to magnetic beads. We tested the applicability of QIAamp Circulating Nucleic Acid kit (Qiagen) columns for the extraction of RNA from cell lysates and were able to demonstrate a high degree of precision (Figure 3c). This is in line with a previous study comparing six commercially available extraction methods, where the same type of columns resulted in the highest recovery of spiked DNA in whole blood [26]. Additionally, the data presented here shows that the degree of sensitivity for mRNA detection is highly dependent on the number of transcripts expressed by the targeted cell type. The COLO205 cells expressed considerably higher levels of both targeted genes, as compared to SW480 cells, which enabled a clearer detection of e.g., 25 cancer cells spiked into 3 mL blood and captured with rVAR2 (Figure 4b,c). While CKMT1A could potentially provide a more sensitive target for detection of rare cells in a WBC background, the analysis of healthy controls with no cancer cells spiked in revealed a considerable level of amplification within non-target cells (Figure 4e). Thus, the increased sensitivity in this case was followed by a compromise in the analytical specificity suggesting that assay validation is required for each chosen transcripts. In addition, the presence of WBC cDNA did affect the amplification efficiency of the qPCR reaction at low target strand concentrations (Figure 3b,c). Therefore, possible strategies for reducing the level of WBC contamination after bead pull-down can be further evaluated.

## 5. Conclusions

This study, together with several previous studies, have strived to provide an optimized assay characterized by a high degree of sensitivity without compromising specificity for the efficient detection of CTCs from early-stage colorectal cancer patients. Here we demonstrate that tissue-specific transcripts can be detected from as little as 10 cells spiked into 3 mL blood after rVAR2-based capture by using the LQAS technology. The PCR workflow successfully met the criteria for analytical validation, enabling the onward movement into clinical feasibility studies. Whether the colon-specific markers tested here will provide sensitive detection of colorectal CTCs from early-stage cancer patients or whether other relevant markers should be included remains to be tested with validation on clinical samples.

## Data Availability

The data presented in this study are available on request from the corresponding author.

## Supplementary Materials

The following are available online at https://www.mdpi.com/article/10.3390/cancers13235881/s1, Figure S1: CASC3 expression, Figure S2: Standard curves, Figure S3: CASC3 expression after capture.
