# Supplementary material for: Detection of VAR2CSA-Captured Colorectal Cancer Cells from Blood Samples by Real-Time Reverse Transcription PCR
Source: Cancers (Basel). 2021 Nov 23;13(23):5881. doi: 10.3390/cancers13235881 (PMC8657034; doi:10.3390/cancers13235881)
Supplement: Supplementary file 1 [file cancers-13-05881-s001.zip › cancers-1440587-supplementary.pdf]

Supplementary figure S1: CASC3 expression

a

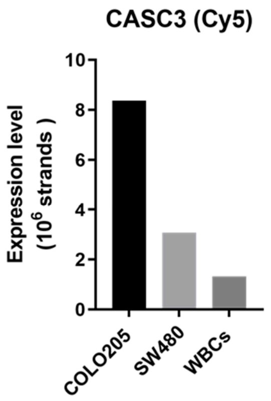

(**a**) CASC3 expression in COLO205 (1,000 cells), SW480 (1,000 cells), WBCs (100,000 cells). The graph diplays the number of detected strands of CASC3 from the cell lysates measured by qPCR following RNA extraction, RT-PCR, and pre-amplification.

Supplementary figure S2: Standard curves

a

USH1C (FAM)

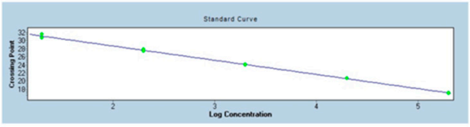

b

CKMT1A (HEX)

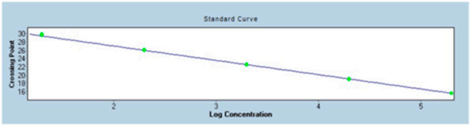

c

CASC3 (Cy5)

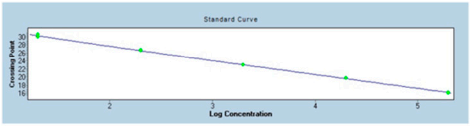

Standard curves for each primer target were included in every qPCR run to enable absolute quantification of strands. Calibrator DNA was diluted 10-fold ranging from 200,000 to 20 strands/reaction. Standard curves were generated using the LightCycler® 480 software by plotting the crossing point (Cp) value against the log(concentration). (**a**) USH1C primers, (**b**) CKMT1A primers, (**c**) CASC3 primers.

Supplementary figure S3: CASC3 expression after capture

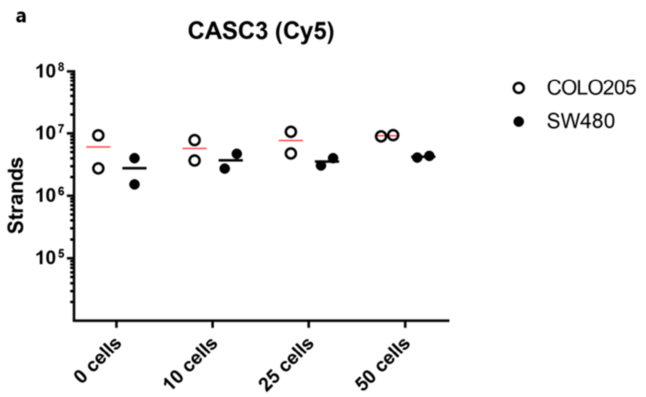

(**a**) Expression of CASC3 control gene. Each dot represent a 3 mL blood sample spiked with a number of COLO205 cells (closed circles) or SW480 cells (open circles) as indicated on the x-axis. Following rVAR2 capture the cells were lysed, RNA extracted, and RT-PCR and preamplification were performed. The graph displays the number of CASC3 strands detected by qPCR.
